# Long-term safety and tolerability of ambrisentan treatment for pediatric patients with pulmonary arterial hypertension: An open-label extension study

**DOI:** 10.1007/s00431-024-05446-1

**Published:** 2024-02-16

**Authors:** Dunbar Ivy, Maurice Beghetti, Ernesto Juaneda-Simian, Ramiya Ravindranath, Mary Ann Lukas, Sandra Machlitt-Northen, Nicola Scott, Jun Narita, Rolf M. F. Berger

**Affiliations:** 1 Pediatric Cardiology, Children’s Hospital Colorado, Aurora, CO USA; 2 Pediatric Cardiology Unit, University Children’s Hospital HUG, Pulmonary Hypertension Program HUG, University of Geneva, Geneva, Switzerland; 3 Pediatric Cardiology, Department of Cardiology, Hospital de Niños de la Santísma Trinidad, Córdoba, Argentina; 4 Biostatistics (R&D), GSK, Bangalore, India; 5 Respiratory/Immunology Clinical Research, GSK, 1250 S Collegeville Rd, Collegeville, Philadelphia, PA 19426 USA; 6 Clinical Science, GSK Medicines Research Centre, Stevenage, UK; 7 Global Safety, GSK, Brentford, Middlesex UK; 8 Pediatrics, Osaka University Graduate School of Medicine, Osaka, Japan; 9 Center for Congenital Heart Diseases, Pediatric Cardiology, Beatrix Children’s Hospital, University Medical Center Groningen, University of Groningen, Groningen, The Netherlands

**Keywords:** Ambrisentan, Endothelin receptor antagonist, Open-label extension, Pediatric PAH

## Abstract

This open-label, extension study assessed long-term safety, tolerability, and efficacy of ambrisentan in a pediatric population (age 8– < 18 years) with pulmonary arterial hypertension (PAH). Following completion of a 6-month, randomized study, participants entered the long-term extension at individualized ambrisentan dosages (2.5/5/7.5 or 10 mg/day). Safety assessments included adverse events (AEs), AEs of special interest, and serious AEs (SAEs); efficacy outcomes included 6-min walking distance (6MWD) and World Health Organization functional class (WHO FC). Thirty-eight of 41 (93%) randomized study participants entered the extension; 21 (55%) completed (reaching age 18 years). Most participants received concomitant phosphodiesterase-5 inhibitors (*n* = 25/38, 66%). Median ambrisentan exposure was 3.5 years. Most participants experienced ≥ 1 AE (*n* = 34/38, 89%), and 21 (55%) experienced SAEs, most commonly worsening PAH (*n* = 3/38, 8%), acute cardiac failure, pneumonia, or anemia (*n* = 2/38; 5% each); none considered ambrisentan-related. Seven participants (18%) died, with recorded reasons (MedDRA preferred term): cardiac failure (*n* = 2), PAH (*n* = 2), COVID-19 (*n* = 1), acute right ventricular failure (*n* = 1), and failure to thrive (*n* = 1); median time to death: 5.2 years. Anemia and hepatotoxicity AEs were generally mild to moderate and did not require ambrisentan dose adjustment. Assessed at study end in 29 participants (76%), mean 6MWD improved by 17% (standard deviation: 34.3%), and all (29/29, 100%) had improved or unchanged WHO FC.

*Conclusion*: Long-term weight-based ambrisentan dosing, alone or combined with other PAH therapies in children with PAH aged 8– < 18 years, exhibited tolerability and clinical improvements consistent with prior randomized study results.

*Trial registration*: NCT01342952, April 27, 2011.

**Table:** 

**What is Known:** *•* *The endothelin receptor antagonist, ambrisentan, is indicated for treatment of pulmonary arterial hypertension (PAH). Previous studies have shown similar efficacy and tolerability in pediatric patients as in adults.*	
**What is New:** *• This open-label extension study assessed the long-term use of ambrisentan in pediatric patients (8–<18 years) with PAH, most of whom were also receiving recommended background PAH treatment. * *• Weight-based dosing of ambrisentan, given alone or in combination with other PAH therapies, was well tolerated with clinical improvements consistent with prior randomized study results.*

**Supplementary Information:**

The online version contains supplementary material available at 10.1007/s00431-024-05446-1.

## Introduction

Pulmonary arterial hypertension (PAH) is a rare, debilitating disease characterized by pulmonary vascular obstruction and vasoconstriction resulting in a progressive increase in pulmonary vascular resistance (PVR), leading to right ventricular failure and death [1, 2]. PAH may present at any age, including infants and children [2]. Although many features are shared between pediatric and adult PAH, epidemiology, etiology, and genetic backgrounds differ [2]. Estimates of point prevalence are 4.4/million children for idiopathic PAH and 15.6/million children for congenital heart disease-associated PAH [3].

Therapeutic interventions for PAH include prostanoids (reviewed in [4]), phosphodiesterase type 5 inhibitors (PDE-5i), and endothelin receptor antagonists (ERA) [1]. Endothelin, a potent vasoconstrictor, is overexpressed in the lungs of individuals with PAH [5], contributing to adverse pulmonary vascular remodeling [6]. ERA efficacy for PAH (World Health Organization functional class [WHO FC] II and III) is supported by a systematic review of 17 randomized controlled trials involving 3322 adults and children aged ≥ 2 years [1].

Ambrisentan, an ERA highly selective for endothelin receptor type A, is indicated for treatment of adults with PAH of WHO FC II to III to improve exercise capacity and delay clinical worsening [7, 8]. In Europe, UK, and Japan, ambrisentan is also indicated for children and adolescents (8 to < 18 years of age) with PAH of WHO FC II to III, including use in combination treatment in Europe [7, 9, 10]. For pediatric PAH, recommended treatment algorithms based on risk stratification and treatment response have been extrapolated from adults and refined using results from observational studies in children with PAH [2].

In a 6-month, randomized, open-label, phase 2b trial (NCT01332331) investigating the safety, tolerability, and efficacy of ambrisentan in a pediatric PAH population (8 to < 18 years) [11], ambrisentan was well tolerated, and adverse events (AEs) and efficacy were consistent with findings in adults with PAH. The current study (NCT01342952) was an open-label, long-term extension of the randomized study; the primary objective was to continue to monitor the safety, tolerability, and efficacy of ambrisentan.

## Materials and methods

### Study design and treatment

Details of the randomized study have been published [11]. Participants were randomized to high- or low-dose ambrisentan (relative to body weight) as oral tablets for 24 weeks: 2.5 mg/day (≥ 20– < 35 kg) or 5.0 mg/day (≥ 35 kg) for the first 2 weeks and then up-titration to 5 mg/day (≥ 20– < 35 kg), 7.5 mg/day (≥ 35– < 50 kg), or 10 mg/day (≥ 50 kg) in the high-dose group [11].

Participants entering the extension had to complete the 24-week randomized study or meet one of the following criteria: (i) Require additional targeted PAH treatment owing to inadequate current treatment response/worsening of clinical condition before Week 24; (ii) require a baseline dose reduction of targeted PAH treatment after addition of ambrisentan to their treatment regimen; or (iii) if continued ambrisentan treatment was considered warranted by the investigator.

Participants were excluded from the extension study if they: had discontinued ambrisentan; were pregnant/breastfeeding; had severe renal impairment (estimated creatinine clearance < 30 mL/min within 45 days prior); or had clinically significant fluid retention and anemia (investigator assessed). All participants provided written informed consent.

Participants entered the extension study at individually tailored dosages (administered in the randomized study), which they continued or could have adjusted based on investigator judgement (not exceeding 0.25 mg/kg/day). Participants remained in the study for ≥ 6 months beyond the initial 6-month study and continued until reaching 18 years of age (primary reason for study completion). Predefined reasons for early withdrawal included liver chemistry values exceeding threshold criteria, an AE which according to investigator opinion required withdrawal, pregnancy, withdrawal of consent, loss to follow-up, or protocol violation.

## Study assessments and procedures

### Primary outcomes: Long-term safety and tolerability of ambrisentan

Safety was assessed from AEs, AEs of special interest (AESIs [anemia, hepatotoxicity, hypersensitivity, hypotension, male infertility, edema, fluid retention]), serious AEs (SAEs), hematology and clinical chemistry, liver function tests, physical examination, pubertal assessments, time to change in ambrisentan dose or other targeted PAH therapy due to tolerability issues, and vital signs recorded at extension study entry and every 3 or 6 months. Values of potential clinical concern (PCC) were defined as outside the ranges for: hemoglobin of 98–180 g/L (male) and 91–161 g/L (female); hematocrit 0.32–0.54 L/L (male) and 0.29 − 0.51 L/L (female); 100–500 GI/L for platelet count; ≥ 34 µmol/L for total bilirubin; and ≥ 177 µmol/L for creatinine, or ≥ 3 × upper limit of normal (ULN) for alanine aminotransferase (ALT), aspartate aminotransferase (AST), and gamma-glutamyl transferase (GGT).

### Secondary outcomes: Long-term efficacy of ambrisentan

Efficacy outcomes included 6-min walking distance (6MWD), WHO FC change, N-terminal pro-B-type natriuretic peptide (NT-proBNP) levels, and time to clinical worsening (definition and additional secondary outcomes listed in Supplementary Methods).

### Statistical analysis

Due to the nature and the size of this study, formal hypothesis testing was not planned. Safety and efficacy data were therefore summarized descriptively. NT-proBNP data were log transformed and summary measures based on analysis of log-transformed data (geometric mean). Kaplan–Meier estimates were used for survival and time to clinical worsening.

The safety and intention-to-treat (ITT) populations comprised participants who received ≥ 1 ambrisentan dose and included all data collected for participants up until the event of death. Participants were analyzed according to the highest ambrisentan dose received during (safety population) or at the start (ITT population) of the extension study. Analyses of efficacy parameters included participants with an end of study assessment recorded, which comprised 21 participants (55%) who completed the study by reaching age 18, plus 8 participants (21%) who had early study withdrawal (but had a recorded end of study assessment). In the Kaplan–Meier analyses, participants were censored at the date they were lost to follow-up.

## Results

### Participant disposition

Of 41 eligible participants from the randomized study, 38 (93%) (19 [50%] from each of the low- and high-dose randomized groups) from 22 centers in 9 countries enrolled in the extension; 37 (97%) had completed the randomized study and one participant entered for whom continued ambrisentan treatment was considered warranted by the investigator despite randomized study non-completion. Over half (*n* = 21/38, 55%) of participants who consented to enter the extension met the criteria for study completion by reaching the age of 18. Most common reasons for withdrawal (*n* = 17/38, 45%) were investigator discretion (*n* = 7/17, 41%) and AEs (*n* = 6/17, 35%) all of which were fatal SAEs (one additional participant died from PAH after reaching age 18 and completing the study), with two participants (12%) lost to follow-up and two (12%) withdrew consent. Reasons for withdrawal due to investigator discretion included enrollment in another study (*n* = 1/17, 6%), participant/investigator decision following updated pharmacovigilance information (*n* = 2/17, 12%), other family decision/reason (*n* = 2/17, 12%), treatment strengthening (*n* = 1/17, 6%), and no reason given (*n* = 1/17, 6%).

### Participant baseline characteristics

Participant baseline characteristics are summarized in Table 1. On extension study entry, of 26 participants (68%) taking additional background PAH therapy, PDE-5i monotherapy was most common (*n* = 17/38, 45%). Eight additional participants (21%) used a PDE-5i in combination with a prostanoid.

**Table 1:** Baseline participant characteristics (ITT population)

	**Ambrisentan dosing group**				**Total (*N* = 38)**
	**2.5 mg (*N* = 9)**	**5 mg (*N* = 19)**	**7.5 mg (*N* = 5)**	**10 mg (*N* = 5)**	
**Age, years, median (range)**^**a**^	8.0 (8–13)	13.0 (8–16)	12.0 (9–16)	15.0 (14–16)	12.5 (8–16)
**Age stratum, *n* (%)**^**a**^					
8 to 11 years	6 (67)	7 (37)	1 (20)	0	14 (37)
12 to < 18 years	3 (33)	12 (63)	4 (80)	5 (100)	24 (63)
**Sex, n (%)**^**a**^					
Female	7 (78)	9 (47)	4 (80)	5 (100)	25 (66)
Male	2 (22)	10 (53)	1 (20)	0	13 (34)
**Childbearing potential for females, *n* (%)**^**b**^					(*N* = 25)
*n*	7	9	4	5	25 (66)
Pre-menarcheal	6 (86)	6 (67)	2 (50)	0	14 (56)
Potentially able to bear children	1 (14)	3 (33)	2 (50)	5 (100)	11 (44)
**Weight, kg, median (range)**^**b**^	29.6 (25.2–37.0)	38.0 (20.1–70.8)	39.8 (36.7–50.0)	58.7 (50.4–77.2)	37.5 (20.1–77.2)
**Weight category, *n* (%)**^**b**^					
20 to < 35 kg	7 (78)	9 (47)	0	0	16 (42)
35 to < 50 kg	2 (22)	5 (26)	4 (80)	0	11 (29)
≥ 50 kg	0	5 (26)	1 (20)	5 (100)	11 (29)
**Etiology of PAH, *n* (%)**^**a**^					
Idiopathic	4 (44)	14 (74)	4 (80)	2 (40)	24 (63)
Persistent PAH despite surgical repair^**c**^	3 (33)	3 (16)	1 (20)	1 (20)	8 (21)
Secondary to connective tissue disease^**c**^	2 (22)	1 (5)	0	1 (20)	4 (11)
Familial^**c**^	0	1 (5)	0	1 (20)	2 (5)
**Duration of PAH, years, median (range)**^**a**^	1.8 (0–7.7)	1.2 (0–11.5)	0 (0–0.8)	5.5 (2.0–11.1)	1.4 (0–11.5)
**WHO FC, *n* (%)**^**b**^					
I	4 (44)	2 (11)	3 (60)	0	9 (24)
II	5 (56)	12 (63)	1 (20)	4 (80)	22 (58)
III	0	4 (21)	1 (20)	1 (20)	6 (16)
IV	0	1 (5)	0	0	1 (3)
**6MWD, m, mean (SD)**^**b**^	485.4 (75.6)	464.5 (125.7)^**d**^	538.0 (119.2)	465.8 (99.3)	479.7 (109.7)
**NT-proBNP, ng/L, geometric mean (log SD)**^**b,e**^	245.8 (0.40)	169.1 (1.37)	66.9 (0.58)	186.9 (1.84)	160.6 (1.27)
**Ongoing background (non-ERA) PAH therapy, *n* (%)**^**b**^					
**Any medication**	7 (78)	12 (63)	3 (60)	4 (80)	26 (68)
** PDE-5i (monotherapy)**	4 (44)	6 (32)	3 (60)	4 (80)	17 (45)
Sildenafil	4 (44)	5 (26)	3 (60)	4 (80)	16 (42)
Sildenafil citrate	0	1 (5)	0	0	1 (3)
** Prostanoid (monotherapy)**	0	1 (5)	0	0	1 (3)
Epoprostenol sodium	0	1 (5)	0	0	1 (3)
** PDE-5i and prostanoid in combination**	3 (33)	5 (26)	0	0	8 (21)
Sildenafil citrate + epoprostenol sodium	0	2 (11)	0	0	2 (5)
Tadalafil + beraprost sodium	1 (11)	1 (5)	0	0	2 (5)
Sildenafil + iloprost	0	1 (5)	0	0	1 (3)
Sildenafil citrate + beraprost sodium	1 (11)	0	0	0	1 (3)
Sildenafil citrate + treprostinil	1 (11)	0	0	0	1 (3)
Tadalafil + treprostinil sodium	0	1 (5)	0	0	1 (3)

*6MWD* 6-min walking distance, *ERA* endothelin receptor antagonist, *NT-proBNP* N-terminal pro-B-type natriuretic peptide, *PAH* pulmonary arterial hypertension, *PDE-5i* phosphodiesterase type 5 inhibitor, *SD* standard deviation, *WHO FC* World Health Organization functional class

^a^At randomized study baseline [11]

^b^At extension study entry

^c^Considered non-idiopathic

^d^*n* = 18

^e^*n* = 5, 12, 4, 5, and 26 for the 2.5 mg, 5 mg, 7.5 mg, 10 mg, and total groups, respectively

### Dosing and exposure

At extension study start, *n* = 19/38 participants (50%), were receiving 5 mg/day ambrisentan. Approximately two-thirds of participants (*n* = 25/38, 66%) continued through the extension study at the ambrisentan dosage assigned during the randomized study, without further up- or down-titration. Participant compliance to treatment was demonstrated at 97.9% of study visits.

Exposure from initial dosing in the randomized study ranged from 3.3 months (100 days) to 10.0 years (3640 days). Median total ambrisentan exposure was 3.5 years (1292 days) and was similar across all dose groups (range: 3.5–4.3 years) with no apparent trend regarding duration of exposure and dosing group. Median time to ambrisentan dose change (or other targeted therapeutic agents) due to tolerability was 393, 469, 354, and 1462 days in the 2.5, 5, 7.5, and 10 mg dose groups, respectively.

### Safety

Most participants experienced ≥ 1 AE (*n* = 34/38, 89%) (Table 2), most commonly upper respiratory tract infection (*n* = 11/38, 29%) and nasopharyngitis (*n* = 9/38, 24%). AEs most frequently considered treatment-related by the investigator were headache (*n* = 3/38, 8%), gastroenteritis (*n* = 2/38, 5%), and anemia (*n* = 2/38, 5%).

**Table 2:** Most common AE and SAE reported during the open-label extension study (safety population)

**Preferred term,** ***n*** **(%)**	**Ambrisentan dosing group**				**Total (*N*** **= 38)**
	**2.5 mg (*N* = 4)**	**5 mg (*N* = 16)**	**7.5 mg (*N* = 6)**	**10 mg (*N* = 12)**	
**Any AE**^**a**^	4 (100)	13 (81)	6 (100)	11 (92)	34 (89)
Upper respiratory tract infection	2 (50)	3 (19)	4 (67)	2 (17)	11 (29)
Nasopharyngitis	0	5 (31)	1 (17)	3 (25)	9 (24)
Headache	0	3 (19)	2 (33)	2 (17)	7 (18)
Anemia	0	2 (13)	0	4 (33)	6 (16)
Pharyngitis	1 (25)	3 (19)	0	2 (17)	6 (16)
Pyrexia	0	2 (13)	2 (33)	2 (17)	6 (16)
Gastroenteritis	1 (25)	2 (13)	0	2 (17)	5 (13)
Influenza	0	3 (19)	1 (17)	1 (8)	5 (13)
Nausea	0	2 (13)	2 (33)	1 (8)	5 (13)
Oropharyngeal pain	0	3 (19)	1 (17)	1 (8)	5 (13)
Epistaxis	0	1 (6)	2 (33)	1 (8)	4 (11)
Pain in jaw	0	2 (13)	2 (33)	0	4 (11)
PAH^b^	0	0	1 (17)	3 (25)	4 (11)
Vomiting	1 (25)	2 (13)	1 (17)	0	4 (11)
**Any SAE**^**c**^	2 (50)	7 (44)	4 (67)	8 (67)	21 (55)
PAH^b^	0	0	0	3 (25)	3 (8)
Cardiac failure acute	0	2 (13)	0	0	2 (5)
Pneumonia	0	1 (6)	1 (17)	0	2 (5)
Anemia	0	1 (6)	0	1 (8)	2 (5)

*AE* adverse events, *PAH* pulmonary arterial hypertension, *SAE* serious adverse event

^a^Reported by ≥ 10% of participants

^b^Included “deterioration of PAH” and “PAH, clinical worsening.” Subjects may have reported more than one event

^c^Reported by more than one participant. SAEs reported by a single participant included: acute right ventricular failure, atrioventricular block complete, atrioventricular block first degree, conduction disorder, right ventricular failure, supraventricular tachycardia, wandering pacemaker, appendicitis, COVID-19, influenza, myringitis, otitis media acute, otitis media chronic, sinusitis, hyperventilation, pulmonary hemorrhage, pulmonary hypertension, disseminated intravascular coagulation, complication associated with device, illness, non-cardiac chest pain, gastric hemorrhage, vomiting, autoimmune lymphoproliferative syndrome, alanine aminotransferase increased, failure to thrive, scoliosis, migraine, dysmenorrhea, hypotension

Twenty-one out of 38 enrolled participants (55%) experienced ≥ 1 SAE, most commonly worsening PAH (*n* = 3/38, 8%), acute cardiac failure, pneumonia, and anemia (*n* = 2/38, 5% each) (Table 2); none were considered ambrisentan-related. All non-fatal SAEs resolved during the study except for pulmonary hemorrhage, gastric hemorrhage, and disseminated intravascular coagulation, all in the same 17.5-year-old participant who died from COVID-19, diagnosed before onset of other SAEs. No dosage adjustments were required because of SAEs, except for one participant in the ambrisentan 2.5 mg dose group who had SAEs of increased ALT, complete atrioventricular block, and hypotension. This participant met protocol-defined liver chemistry stopping criteria for ALT elevation (133.0 IU/L) that was ≥ 3 × ULN (≥ 30 IU/L), occurred approximately 2 years following the first dose of ambrisentan (Day 781), and was reported as severe. Total bilirubin remained within or low of the normal range throughout this study. The other two contemporaneous SAEs, that were severe in intensity (complete atrioventricular block and hypotension), also led to temporary dose interruption, and all events resolved within 23 days. The ALT elevation did not require intervention, and the participant did not develop fulminant liver failure, with the episode of complete heart block during cardiac catheterization considered a contributing factor. Following rechallenge with ambrisentan, the event did not recur.

Seven deaths (*n* = 7/38, 18%) occurred during extension study participation (Table 3); none of the fatal SAEs were considered ambrisentan-related by the investigator. There were two fatal SAEs of acute cardiac failure (both 5 mg dose group), two of PAH (both 10 mg dose group), and one each of COVID-19 (5 mg dose group), acute right ventricular failure (5 mg dose group), and failure to thrive (7.5 mg dose group). Median time to death was 5.2 years. Kaplan–Meier survival estimates were 94.7% at 3 years and 92.1% at 4 years after treatment initiation and 81.6% at study end.

**Table 3:** SAEs leading to death during the open-label extension study (safety population)

	**Ambrisentan dosing group at end of study**						
	**5 mg/day (*N* = 19)**				**7.5 mg/day (*N* = 5)**	**10 mg/day (N = 5)**	
**Fatal SAE by preferred term**	Cardiac failure acute/acute cardiac decompensation	Cardiac failure acute/acute cardiac decompensation	COVID-19	Acute right ventricular failure/acute right heart failure	Failure to thrive^a^	PAH/deterioration of PAH^b^	PAH/deterioration of PAH^c^
**Demographics at study entry**							
Age (years)	9	8	9	9	12	16	9
Sex	Female	Female	Female	Female	Female	Female	Male
Race	White	White	White	White	White	White	White
Weight (kg)	26.6	20.1	36.0	24.4	28.0	56.2	40.0
**Disease history**							
Diagnosis	Idiopathic	Idiopathic	Idiopathic	Familial	Idiopathic	Idiopathic	Idiopathic
Duration of PAH (years)	1.7	0	6.4	8.4	8.3	2.0	0.8
Baseline WHO FC	II	II	II	III	II	III	III
Baseline NT-proBNP, ng/L	252.0	5738.0	169.0	460.0	453.0	1657.0	238.0
**Date of death**	13 Apr. 2016	29 Sept. 2012	22 Jul. 2020	5 Jun. 2016	25 Jul. 2017	30 Aug. 2014	24 Jun. 2019
Study day of death	1884	101	2829	1293	1909	473	2232
Exposure to ambrisentan (study day of final dose)	1884	100	2829	1292	1896	440	2230
**Last recorded WHO FC at time of death**	III	IV	II	III	III	IV	III
Day of WHO FC recording	1723	89 (extension study entry visit)	2722	1177	1668	469 (follow-up visit)	2191
**Last recorded NT-proBNP, ng/L**	3415.0	5738.0	44.0	630.0	742.0	9013.0	3526.0
Day of last NT-proBNP recording	1723	Baseline	2557	1177	907	469 (follow-up visit)	2191
**Concomitant PAH therapy**							
Ingredient (total daily dosage)	Sildenafil (60 mg)	Sildenafil (10 mg)	None recorded	Sildenafil (75 mg)^d^	Sildenafil (60 mg)	Sildenafil (30 − 60 mg)^e^	Sildenafil (30–60 mg)^f^
Study day started	1406	91		Pre-study (− 3017)	Pre-study (− 2489)	Pre-study	Pre-study
Study day stopped	Ongoing at time of death	101 (day of death)		Ongoing at time of death	Ongoing at time of death	Ongoing at time of death	2230 (2 days before death)
Ingredient (total daily dosage)	Treprostinil (10 ng/kg/min)	Iloprost (0.5 mg/kg/min)			Epoprostenol (116 − 154 ng/kg/min)	Bosentan (125 mg)	
Study day started	1308	87			Pre-study (− 3001)	441 (post-study treatment)	
Study day stopped	Ongoing at time of death	101 (day of death)			Ongoing at time of death (several dose changes and a change in brand)	Ongoing at time of death	

*NT-proBNP* N-terminal pro-B-type natriuretic peptide, *PAH* pulmonary arterial hypertension, *SAE* serious adverse event, *WHO FC* World Health Organization functional class

^a^Participant was in the 5 mg/day group, was up-titrated to 7.5 mg/day on study day 632, and remained on 7.5 mg/day to final dose

^b^Participant was 18 at the time of death and had completed the main part of the extension study

^c^Participant was in the 7.5 mg/day group, was up-titrated to 10 mg/day on study day 1365, and remained on 10 mg/day to final dose

^d^Participant also took aspirin, spironolactone, and digoxin

^e^Participant also took hydrochlorothiazide + triamterene, torasemide, and furosemide

^f^Participant also took captopril, hydrochlorothiazide + triamterene, torasemide, and furosemide

Overall, 20/38 (53%) experienced an AESI, most commonly anemia (*n* = 6/38, 16%) (Table 4). Two participants (*n* = 2/38, 5%) (5 and 10 mg dose groups) experienced moderate anemia events (two considered to be low hemoglobin levels of PCC, one in each participant) that were considered ambrisentan-related. No ambrisentan dosage adjustments were required and anemia resolved in both participants. Six participants (*n* = 6/38, 16%) reported 11 anemia events; all resolved without ambrisentan dosage adjustment, except one moderate and non-serious event, which remained intermittent with no dose adjustment in a participant in the 10 mg dose group who had a history of anemia, hypothyroidism, and chronic sinusitis/epistaxis. Additionally, three participants (*n* = 3/38, 8%) recorded AESIs of iron deficiency that were listed as resolving/not resolved at study end. AESIs related to hepatotoxicity and hepatobiliary disorders were experienced by four participants (*n* = 4/38, 11%) across three dosing groups (2.5, 5, 10 mg); for two of these participants, PCC levels for ALT, AST, total bilirubin, and/or GGT were also reported (in total there were six participants with PCC levels, four did not have a corresponding AESI). All of these AESIs were resolved except for one instance of elevated AST (although not recorded as resolved in the AE report for this single episode, the next AST result taken 11 days later showed a return to within normal range with no concurrent abnormalities for ALT or total bilirubin) and one instance of hepatomegaly (unresolved at time of death from acute cardiac failure). Elevated AST and bilirubin levels (both mild and non-serious) in one participant (10 mg dose group) were considered ambrisentan-related but did not result in dose interruptions or adjustments. All remaining AESIs pertaining to liver function tests and hepatomegaly were not considered ambrisentan-related.

**Table 4:** Most common AESI (reported by more than one participant) reported during the open-label extension study (safety population)

**Preferred term, *n*** **(%)**	**Ambrisentan dosing group**				**Total (*N*** **= 38)**
	**2.5 mg (*N* = 4)**	**5 mg (*N* = 16)**	**7.5 mg (*N* = 6)**	**10 mg (*N* = 12)**	
**Any AESI**	2 (50)	8 (50)	3 (50)	7 (58)	20 (53)
Anemia	0	2 (13)	0	4 (33)	6 (16)
Dermatitis contact	0	1 (6)	1 (17)	1 (8)	3 (8)
Erythema	1 (25)	1 (6)	1 (17)	0	3 (8)
Rash	0	1 (6)	2 (33)	0	3 (8)
Iron deficiency anemia	0	3 (19)	0	0	3 (8)
Rhinitis allergic	0	2 (13)	0	1 (8)	3 (8)
Eczema	0	1 (6)	0	1 (8)	2 (5)
Pruritus	0	1 (6)	0	1 (8)	2 (5)
AST increased	1 (25)	0	0	1 (8)	2 (5)
Edema peripheral	0	1 (6)	0	1 (8)	2 (5)
Conjunctivitis allergic	0	1 (6)	0	1 (8)	2 (5)
Dizziness	0	0	2 (33)	0	2 (5)

*AESI* adverse event of special interest, *AST* aspartate aminotransferase

At randomized study baseline, 40% of female participants (*n* = 10/25) and 38% of male participants (*n* = 5/13) were pre-adolescent. At extension study end, most participants were at later stages of pubertal development. No pregnancies were reported, and no clinically relevant changes from baseline were observed for plasma endocrinology parameters. Physical examination parameters generally remained stable with no clear pattern of change throughout the extension study.

### Impact of ambrisentan on efficacy parameters

**Fig. 1:**
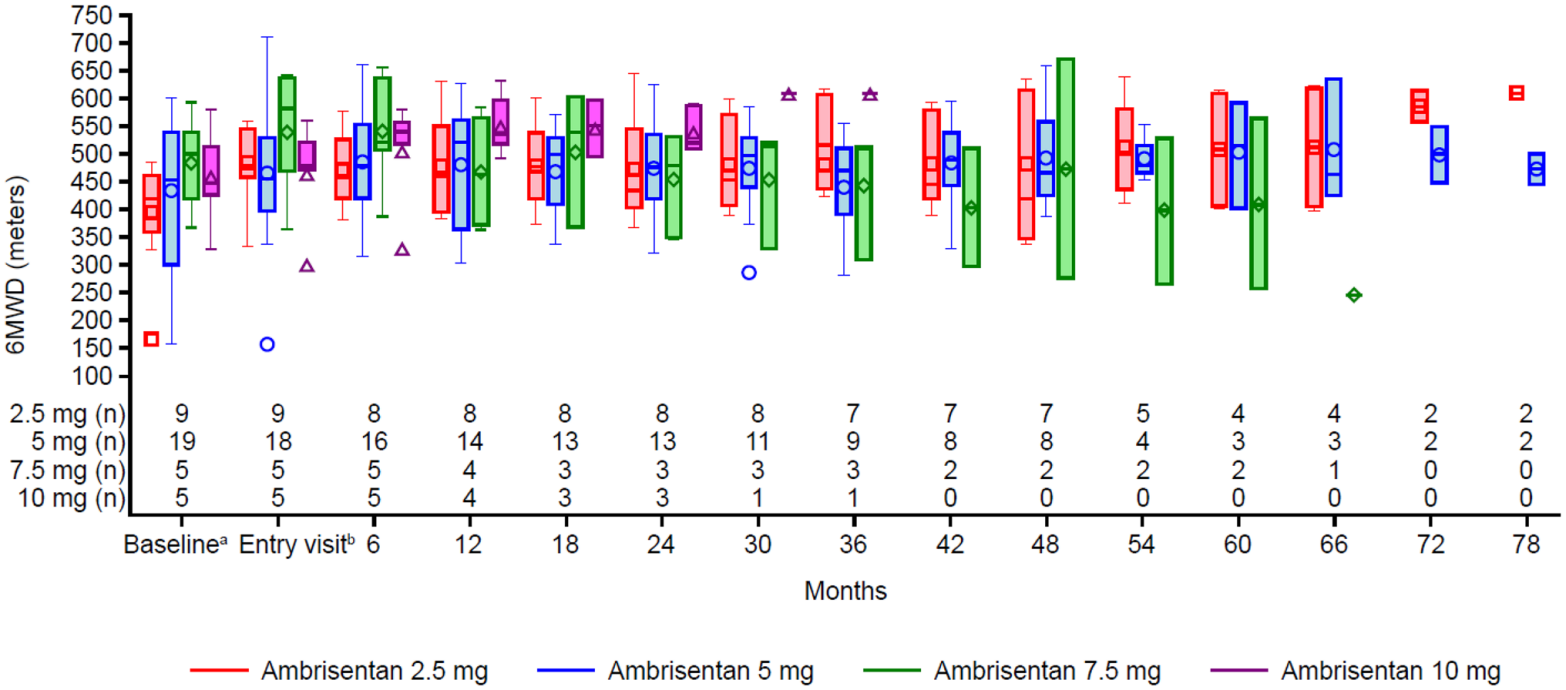
Change in 6MWD from randomized study baseline by study visit (ITT population). ^a^Baseline for the randomized study. ^b^Entry into the open-label extension study. 6MWD, 6-min walking distance; ITT, intention-to-treat

At randomized study baseline, mean 6MWD was 434.4 m (standard deviation [SD] 110.37, *n* = 38), which increased to 479.7 (SD 109.69, *n* = 37) at extension study entry. In 29 of the 38 enrolled participants (76%) with an end of study assessment recorded, 6MWD increased by a mean of 58.4 m (SD 88.15), representing a mean increase of 17.0% (SD 34.3). Figure 1 shows the change in 6MWD by visit and dosage group.

When the last recorded 6MWD was assessed for all 38 participants regardless of completion status, 22 (58%) had achieved a clinically significant improvement in 6MWD (increase in walk distance by ≥ 20 m) from randomized study baseline, with comparable proportions for those with an idiopathic etiology (*n* = 14/24, 58%) or non-idiopathic etiology (*n* = 8/14, 57%) of PAH.

Of 29 participants (76%) with an WHO FC assessment recorded at study end, 13/29 (45%) showed an improvement in WHO FC, 16/29 (55%) remained unchanged, and there were no deteriorations. Most improved by one WHO FC category (11/13, 85%), and two participants improved by two categories.

**Fig. 2:**
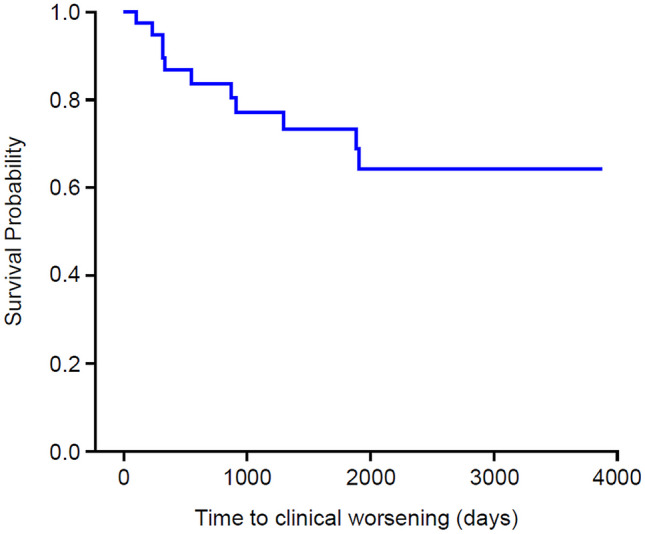
Kaplan–Meier survival curve of time to first clinical worsening of PAH (ITT population). Time to clinical worsening of PAH was defined as time to first occurrence of the following: all-cause mortality or placement on active list for lung transplant and/or atrial septostomy; hospitalization due to deterioration of PAH; addition of another targeted PAH therapy due to deterioration of clinical condition; dose modification of ambrisentan or other targeted PAH therapy due to deterioration of clinical condition; and PAH-related deterioration. ITT, intention-to-treat; PAH, pulmonary arterial hypertension

Overall, 11/38 participants (29%) experienced clinical worsening of PAH based on ≥ 1 criterion, with an event rate for clinical worsening estimated as 8.6/100 patient-years. Median time from randomized study baseline to clinical worsening of PAH was approximately 1.5 years (range 3 months to 5.2 years) (Kaplan–Meier analysis; Fig. 2).

**Fig. 3:**
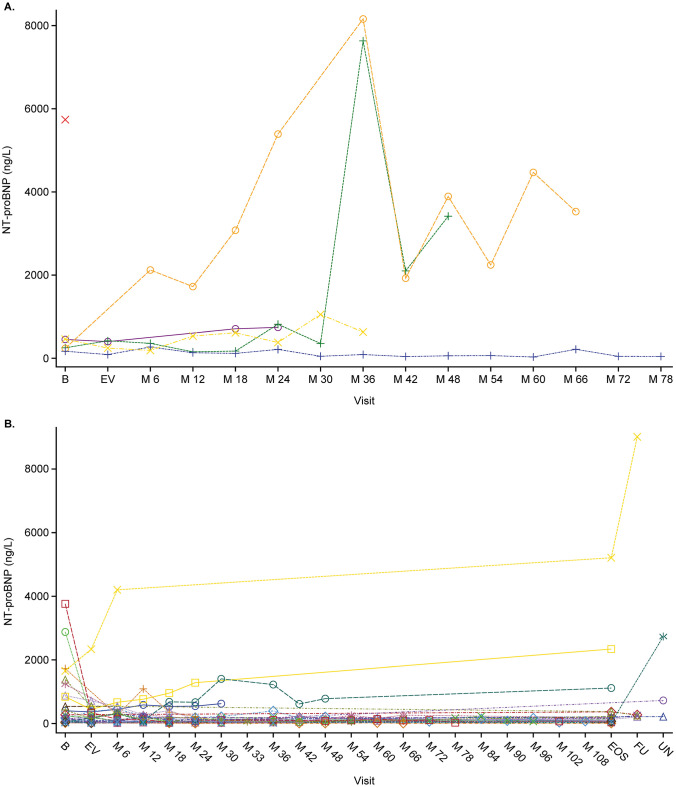
NT-proBNP levels over time in **A** patients who died and **B** patients who did not die. B, baseline; EV, entry visit; M, months; EOS, end of study; FU, follow-up; NT-proBNP, N-terminal pro-B-type natriuretic peptide; UN, unscheduled

**Fig. 4:**
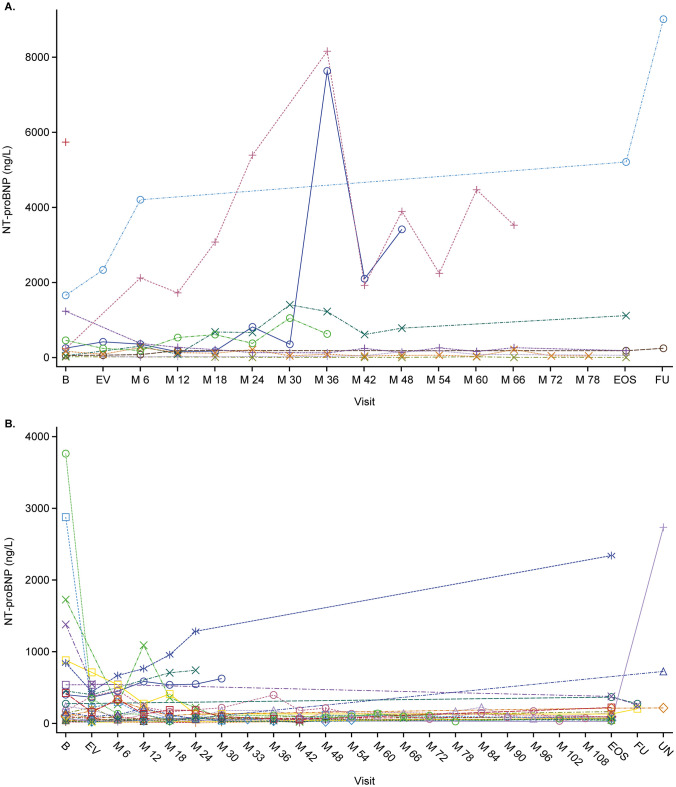
NT-proBNP levels over time in **A** patients who had worsening of PAH and **B** patients who did not have worsening of PAH. B, baseline; EV, entry visit; M, months; EOS, end of study; FU, follow-up; NT-proBNP, N-terminal pro-B-type natriuretic peptide; PAH, pulmonary arterial hypertension; UN, unscheduled

The geometric mean value for NT-proBNP was 224.1 ng/L (SD 1.50) at randomized study baseline for the 37 participants (97%) with available data. For the 25 participants (66%) with a value recorded at the end of study visit, there was a mean decrease of 36.8% (SD 1.68). NT-proBNP levels were also evaluated in all 38 participants as to being above or below 1200 ng/L (a level indicated as a viable treatment goal in pediatric patients to improve survival outcomes), including the baseline visit in the 24-week randomized study prior to initiation of ambrisentan. Among the 5 participants (13%) whose single NT-proBNP level > 1200 ng/L was recorded at the randomized study baseline prior to ambrisentan initiation, 1 had an event of PAH worsening during the extension study, whereas 5/6 participants (83%) who had ≥ 1 recorded NT-proBNP level > 1200 ng/L subsequent to that baseline had an event of PAH worsening or death. Of the 27 participants (71%) with no NT-proBNP value > 1200 ng/L recorded, 6/27 (22%) had PAH worsening or death. Figures 3 and 4 display NT-proBNP levels over time and by whether patients had/did not have events of PAH worsening or death.

## Discussion

This long-term open-label extension study showed that ambrisentan, administered alone or in combination with ongoing PAH therapies (including PDE-5i therapies), for a median duration of 3.5 years (range: 0.3–10 years), was well tolerated by participants 8 to < 18 years of age. AESIs of anemia and hepatotoxicity were generally mild to moderate in intensity and non-serious, did not lead to ambrisentan dosage adjustments or discontinuation, and resolved for all but one instance of anemia and two instances of hepatotoxicity (one elevated AST not recorded as resolved in the AE report but with laboratory evidence of resolution after 11 days, and one hepatomegaly unresolved at time of death due to acute cardiac failure). Improvements in exercise capacity, WHO FC, and NT-proBNP observed in the initial randomized study continued throughout the extension analyzed for the participants with an end of study assessment.

The most common AEs were upper respiratory tract infection, nasopharyngitis, and headache, similar to the randomized study and ambrisentan product labeling for adults [7, 8, 11]. No non-fatal AEs led to discontinuation of ambrisentan or study withdrawal. SAEs resulted in the death of seven participants, and all except one fatal SAE of COVID-19 were associated with the underlying disease and its progression; investigators considered none to be ambrisentan-related.

Clinically relevant improvements in exercise capacity observed in the randomized study continued with long-term ambrisentan treatment; more than half of the 38 participants showed an increase in walk distance by ≥ 20 m from randomized study baseline to their last observation, which did not seem to be driven by idiopathic or non-idiopathic etiology. Due to the long follow-up duration, age-related improvements in 6MWD (due to growth) may have contributed to these findings. As the improvements in the open-label extension were consistent with those observed in the shorter randomized trial, it is likely that treatment-related improvements were the driving factor.

Among participants who had WHO FC assessed at study end, all showed either improvement or no change with no deteriorations. Similarly, among participants who had available NT-proBNP data at an end of study visit (i.e., those who completed the study at age 18, or who withdrew but had that visit performed), the mean NT-proBNP level decreased by − 36.8%. These data should be interpreted with caution as NT-proBNP values varied considerably over the study, and the potential for survivor bias cannot be ruled out.

Tolerability and clinical benefit of long-term ambrisentan treatment were apparent. Most participants maintained the same dosage for the duration of treatment in the randomized study and open-label extension, and participants demonstrated compliance to their treatment regimen at 98% of study visits. Following the pivotal ARIES studies investigating ambrisentan at 2.5, 5, and 10 mg/day in adults with PAH [12], the first report in children with PAH indicated that ambrisentan pharmacodynamics and safety were comparable with adults [13]. More recently, a study employing pharmacokinetic modeling and exposure–response analysis of ambrisentan comparing pediatric data derived from the randomized study with adult data from several phase I–III trials concluded that ambrisentan response profiles are similar when weight-based dosing is used for children (8 to < 18 years of age; 2.5 or 5 mg/day for 2 weeks, then the option to up-titrate to 5, 7.5, or 10 mg/day dependent on body weight) [14], and the results from this study further support this approach.

Owing to small sample sizes, study results should be interpreted carefully. Accounting for the inherent challenges associated with conducting pediatric trials, particularly for rare diseases like PAH, this study represents one of the longest investigations to date of an ERA in pediatric patients [2, 15]. This study permitted concomitant use of PDE-5i therapy (e.g., sildenafil), which is in line with current treatment guidelines but was not permitted in a previous long-term pediatric study of the ERA bosentan [15]. Entry criteria also permitted recruitment of a heterogeneous group, which largely comprised participants with idiopathic or persistent PAH despite surgical repair and who were naïve to treatment or took different treatment regimens. The etiology for participants was in line with expectations for pediatric presentation of PAH.

## Conclusion

This extension study provides valuable information for patients, healthcare providers, and regulators on long-term ambrisentan use in a pediatric population with PAH, the majority of whom were receiving concomitant PDE-5i therapy as per current recommendations [2]. No new safety concerns were reported, and efficacy assessments were consistent with the randomized study. There results bolster support for weight-based ambrisentan dosing in pediatric patients aged 8 to < 18 years (2.5 or 5 mg/day for 2 weeks, then optional up-titration to 5, 7.5 or 10 mg/day dependent on body weight).

### Supplementary Information

Below is the link to the electronic supplementary material.

Supplementary file1 (DOCX 64 KB)

## Data Availability

Anonymized individual participant data and study documents can be requested for further research from https://www.gsk-studyregister.com/en/. Additional data are provided in the supplement available online.

## Abbreviations

6MWD: 6-min walking distance
AE: Adverse event
AESI: Adverse event of special interest
ALT: Alanine aminotransferase
AST: Aspartate aminotransferase
ERA: Endothelin receptor antagonist
GGT: Gamma-glutamyl transferase
ITT: Intention-to-treat
NT-proBNP: N-terminal pro-B-type natriuretic peptide
PAH: Pulmonary arterial hypertension
PCC: Potential clinical concern
PDE-5i: Phosphodiesterase type 5 inhibitor
PVR: Pulmonary vascular resistance
SAE: Serious adverse event
SD: Standard deviation
ULN: Upper limit of normal
WHO FC: World Health Organization functional class
