# Supplementary material for: Long-term safety and tolerability of ambrisentan treatment for pediatric patients with pulmonary arterial hypertension: An open-label extension study
Source: Eur J Pediatr. 2024 Feb 16;183(5):2141–53. doi: 10.1007/s00431-024-05446-1 (PMC11035402; doi:10.1007/s00431-024-05446-1)
Supplement: Supplementary file 1 — Supplementary file1 (DOCX 64 KB) [file 431_2024_5446_MOESM1_ESM.docx]

##### **Supplementary Methods**

The time to clinical worsening of pulmonary arterial hypertension (PAH) was defined as the time to first occurrence of:

- All-cause mortality or placement on active list for lung transplant and/or atrial septostomy;
- Hospitalization due to deterioration of PAH;
- Addition of another targeted PAH therapy due to deterioration of clinical condition;
- Dose modification of ambrisentan or other targeted PAH therapy due to deterioration of clinical condition;
- PAH-related deterioration identified by:
  - Increase in World Health Organization functional class;
  - Deterioration in exercise testing (20% decrease in 6-minute walking distance based on 2 consecutive tests 1 week apart
  - Clinical signs/symptoms of right-sided heart failure (i.e., new peripheral edema; increased liver size; ascites; increased jugular venous pressure; pericardial effusion; increased dyspnea).

Additional secondary outcomes:

- All-cause mortality
- Time to addition of another targeted PAH therapeutic agent
- Time to change in dose of ambrisentan or other targeted PAH therapeutic agent
- 10-item Short-Form Health Survey for children
- Missed school days
